# Brain-potential analysis of visual word recognition in dyslexics and typically reading children

**DOI:** 10.3389/fnhum.2014.00474

**Published:** 2014-06-30

**Authors:** Gorka Fraga González, Gojko Žarić, Jurgen Tijms, Milene Bonte, Leo Blomert, Maurits W. van der Molen

**Affiliations:** ^1^Department of Psychology, University of AmsterdamAmsterdam, Netherlands; ^2^Rudolf Berlin CenterAmsterdam, Netherlands; ^3^Department of Cognitive Neuroscience, Maastricht UniversityMaastricht, Netherlands; ^4^Maastricht Brain Imaging Center, Maastricht UniversityMaastricht, Netherlands; ^5^IWAL Institute, AmsterdamNetherlands; ^6^Amsterdam Brain and Cognition, University of AmsterdamAmsterdam, Netherlands

**Keywords:** reading fluency, developmental dyslexia, visual word recognition, event-related potentials, N1, visual attention

## Abstract

The specialization of visual brain areas for fast processing of printed words plays an important role in the acquisition of reading skills. Dysregulation of these areas may be among the deficits underlying developmental dyslexia. The present study examines the specificity of word activation in dyslexic children in 3rd grade by comparing early components of brain potentials elicited by visually presented words vs. strings of meaningless letter-like symbols. Results showed a more pronounced N1 component for words compared to symbols for both groups. The dyslexic group revealed larger left-lateralized, word-specific N1 responses than the typically reading group. Furthermore, positive correlations between N1 amplitudes and reading fluency were found in the dyslexic group. Our results support the notion of N1 as a sensitive index of visual word processing involved in reading fluency.

## INTRODUCTION

Reading involves decoding visual information to access a series of speech sounds, and word meanings. Fluent readers develop visual expertise that allows fast identification, recognition and categorization of letters, and this specialization is proposed to recruit specific cortical areas evolved for visual object recognition (Dehaene and Cohen, 2007). This ability is compromised in individuals diagnosed with developmental dyslexia. Dyslexia is a specific reading disability with a neurobiological origin, persistent symptoms and high prevalence rates ranging from 5 to 10% (Blomert, 2005; Shaywitz and Shaywitz, 2005). It is characterized by dysfluent and inaccurate word recognition, spelling and phonological decoding (Lyon et al., 2003). The lack of fluency seems to be the most persistent feature and typical levels of automaticity in reading are hardly achieved after treatment (Tijms et al., 2003; Shaywitz and Shaywitz, 2008; Benjamin and Gaab, 2012).

Neuroimaging studies explored the neural correlates of both phonological and visual recognition processes during reading. Two posterior neural systems, primarily in the left hemisphere, have been described as particularly important in the development of reading skills (McCandliss et al., 2003). One of these systems is located in the left dorsal parieto-temporal region and involves areas of the superior temporal gyrus, supramarginal gyrus and angular gyrus. This system is related to phonological processing and cross-modal integration of letter and speech sounds (van Atteveldt et al., 2004; Blomert, 2011). The second system, which is the focus of the present study, is located in the ventral left occipito-temporal region, and involves areas in the middle and inferior temporal and occipital gyrus. Within this system, the area located at the left lateral occipito-temporal sulcus has been called the “visual word form area” (VWFA) because of its suggested specialization for printed word recognition (McCandliss et al., 2003; Dehaene and Cohen, 2011).

Longitudinal studies suggest that the left dorsal parieto-temporal system and the left occipito-temporal system interact and play an important role in the development of reading acquisition. Accordingly, some authors proposed a model in which the temporo-parietal system develops earlier and establishes letter-speech sounds (LSS) mappings that later supports the rapid word recognition specialization subserved by the occipito-temporal system (McCandliss and Noble, 2003; Sandak et al., 2004; Blomert, 2011). Most importantly, dysregulation in both the posterior parieto-temporal and occipito-temporal systems for reading have been found in dyslexic adults (Brunswick et al., 1999; Helenius et al., 1999; Simos et al., 2000; Paulesu et al., 2001; Shaywitz and Shaywitz, 2008; Blau et al., 2010).

Electrophysiological studies allow for taking a closer look at the time course of neural responses to print and can provide substantial information regarding the functional aspects of the occipito-temporal system during reading. Studies examining event-related brain potentials (ERPs) yielded two components related to early visual processing of orthographic stimuli. The first is a positive component labeled P1, it peaks between 100 and 150 ms after stimulus onset and it has posterior-occipital topography. P1 has been associated with low-level analysis of word features, including word length and typicality (Assadollahi and Pulvermüller, 2003; Hauk et al., 2006). A second component, labeled N1 or N170, has a negative polarity and peak latencies around 200 ms, and is usually observed at parieto-occipital or occipital sites. Most interestingly, N1 has been related to visual expertise and orthographic processing (Bentin et al., 1999; Maurer et al., 2005), and its sources have been localized in the VWFA (Tarkiainen et al., 1999; Rossion et al., 2003). In addition to P1 and N1, a later positivity (labeled P2 in this study), with latencies around 300 ms and more temporal topographies, has been associated with phonological as well as semantic aspects of stimuli in visual word experiments (Nobre et al., 1994; Landi and Perfetti, 2007).

The N1 component is the main focus of the present study because of its relation to visual processing and VWFA activity. Expertise in the visual processing of different categories of objects is associated with an enhancement of N1 amplitude (Tanaka and Curran, 2001). Interestingly, besides general visual expertise, N1 seems to be particularly sensitive to lexical processing. Larger N1 amplitudes are found for words compared to strings of symbols, shapes, or dots (Bentin et al., 1999; Tarkiainen et al., 1999; Maurer et al., 2005). Moreover, N1 responses appear to be sensitive to word similarity, being larger to letters-like stimuli like pseudofonts compared to stimuli matched on low-level features (Schendan et al., 1998; Tarkiainen et al., 1999; Eulitz et al., 2000). Further, consonant strings and pseudowords usually evoke N1 responses similar to those elicited by words (Bentin et al., 1999). In addition, the N1 specialization for word processing seems to be automatic, and is observed when using tasks that do not require reading (Bentin et al., 1999; Eulitz et al., 2000; Brem et al., 2005). To some extent, N1 responses to words may relate to a more general N1 sensitivity to familiarity. However, evidence supporting the left lateralization of N1 word responses suggests that this may be a special of perceptual expertise. A number of studies have reported left lateralized enhancement of N1 amplitudes to orthographical compared to contrast visual stimuli (Bentin et al., 1999; Rossion et al., 2003; Maurer et al., 2005; Xue and Poldrack, 2007; Maurer et al., 2008). Collectively, these findings suggest that N1 can be used to examine fast and automatic neural responses to print. In view of this evidence, N1 amplitude differences between words vs. symbol strings have been used to provide an index for “visual tuning” for print that is proposed to develop with visual learning during the first years of reading acquisition (Maurer et al., 2008). This is referred to as the “visual tuning” hypothesis.

In a series of ERP studies, Maurer and colleagues compared N1 differences between words vs. strings of icon-like symbols at different stages of reading acquisition in both normal readers and dyslexics (Maurer and McCandliss, 2008; Maurer et al., 2011). The data of normal readers suggested a significant left-lateralized N1 tuning effect that remains relatively stable during the first years of reading acquisition (Maurer et al., 2005). The N1 word-symbol differences in typically reading children were larger for 2nd grade children relative to kindergartners, but leveled off between 2nd grade and 5th grade (Maurer et al., 2011). This pattern of findings was taken to suggest an inverted “U” model of development of visual expertise, in which perceptual learning becomes highly important during the first two or three years of learning to read and then gradually declines as expertise develops. In the same series of studies, the dyslexic children in 2nd grade showed a reduced word vs. symbol difference in N1 amplitude as compared to normal readers. The authors interpreted the reduced word-symbol difference in dyslexics as a lack of visual specialization for print, reflecting a deficit in expertise for rapid word recognition. The N1 amplitude difference between 2nd grade typical readers and dyslexic readers did not reach significance when the groups were compared at 5th grade (Maurer et al., 2011; Hasko et al., 2012). Related ERP studies suggested, however, that this deficit continues to persist in pre-adolescents (Araújo et al., 2012) and adulthood (Helenius et al., 1999; Mahé et al., 2012). Similarly, an fMRI study found differences also in 4th and 5th grade (van der Mark et al., 2009).

The present study further examines the N1 component in dyslexic readers by using an implicit word-reading task and presenting letter-like strings of symbols as contrast. The false font used resembles alphabetic letters but consists of completely novel symbols. Thus we expect this type of symbols to prevent top-down influences from letter representations. In addition, the use of an implicit reading task allows for the assessment of early visual processing, not biased by reading level (Brunswick et al., 1999). This experimental design should demonstrate that N1 amplitude qualifies as a sensitive index of visual specialization for print. Furthermore, the relation between N1 amplitude and reading in typical and dyslexic 3rd grade readers will be assessed with a special emphasis on fluency. Previously, Maurer et al. (2006, 2007) observed a relation between N1 amplitude to word-symbols and reading speed but only when collapsing the typically reading and dyslexic groups. In the present study we will examine the relation between N1 and reading fluency in both groups, separately.

## MATERIALS AND METHODS

### PARTICIPANTS

Third-grade dyslexic children (*N* = 19; 8.97 ± 0.39 years old) were recruited from a nation-wide center for dyslexia in the Netherlands. All of them had a percentile score of 10 or lower on a standard reading test and they participated in the ERP experiment before starting their treatment program at the center. A group of 20 third-grade, typical readers (8.78 ± 0.35 years old) was recruited from several primary schools attended by children with the same sociodemographical background as the dyslexic group (see **Table 1** for group characteristics). They had no history of reading difficulties and had a percentile score of 25 or higher on standard reading tests (see below). All participants were native Dutch speakers, received two and a half years of formal reading instruction in primary education. Children with below average IQ (IQ < 85 on a non-verbal IQ-test), uncorrected sight problems, hearing loss, diagnosis of ADHD or other neurological or cognitive impairments were excluded. The study was approved by the ethics committee of the university and all parents or caretakers signed informed consent before the children participated.

**Table 1:** Descriptive statistics showing reading accuracy and fluency scores.

	Typical Readers	Dyslexics		

	*M (SD)*	*M (SD)*	*p*-value	η^2^
*N*	20	19		
Sex ratio (m:f)	8:12	8:11		
Handedness (L:R)^*^	2:15	3:16		
Age	8.78 (0.35)	8.97 (0.39)	0.122	0.34
					
3DM Word reading – *accuracy*^a^					
HF	99.12 (1.12)	93.75 (4.33)	0.000	0.44
LF	97.25 (3.23)	86.46 (13.52)	0.001	0.24
Pseudo	87.37 (9.65)	69.14 (17.77)	0.000	0.30
Total [T]^b^	49.50 (9.06)	31.05 (10.23)	0.000	0.49
					
3DM Word reading – *fluency* [T]					
HF	52.95 (7.58)	31.68 (6.03)	0.000	0.72
LF	54.65 (9.02)	31.53 (5.92)	0.000	0.70
Pseudo	53.00 (9.44)	29.84 (6.70)	0.000	0.68
Total	53.95 (9.34)	30.68 (4.84)	0.000	0.72
					
One-Minute Test – *fluency* [C]^c^	6.05 (1.76)	2.00 (0.88)	0.000	0.69
Text Reading – *fluency*[T]^**^	54.70 (8.04)	32.94 (5.94)	0.000	0.71
					
3DM Spelling – *accuracy*[T]	50.60 (9.14)	34.37 (5.00)	0.000	0.56
3DM Spelling – *fluency*[T]	54.55 (8.70)	36.68 (6.28)	0.000	0.60
					
Letter-speech sound associations [T]					
L-SS identificacion – *accuracy*	46.95 (7.70)	39.00 (9.08)	0.005	0.19
L-SS discrimination – accuracy^**^	50.20 (9.25)	40.72 (8.04)	0.002	0.24
L-SS identificacion – *fluency*	52.80 (7.08)	41.53 (8.02)	0.000	0.37
L-SS discrimination – fluency^**^	51.10 (8.01)	43.28 (8.61)	0.006	0.19
					
3DM Naming speed scores[T]					
Letters	50.05 (7.13)	37.95 (7.67)	0.000	0.41
Numbers	50.65 (10.92)	38.95 (8.60)	0.001	0.27
Total	49.85 (7.91)	36.84 (8.60)	0.000	0.40

a Raw scores. ^b^T scores (*M* = 50, *SD* = 10). ^c^C scores (*M* = 5, *SD* = 2).

* Data missing for three participants; Typical *n* = 17. **Data missing for one participant; Dyslexics *n* = 18.

### BEHAVIORAL MEASUREMENTS

A series of tests were used to assess the reading skills of the participants. The children took the tests at their school.

Word reading skills were measured using a Dutch version of the *One-minute test* (*Een-Minuut-Test*, EMT; Brus and Voeten, 1973). It is a time-limited test consisting of a list of 116 unrelated words of increasing difficulty and the number of correctly read words within 1 min serves as reading fluency score. Text reading fluency was assessed also using a test consisting of a coherent text of increasing difficulty. The children were asked to read the story out loud within one minute (*Schoolvaardigheidstoets Technisch Lezen*; de Vos, 2007). In addition, the 3DM battery of tests (test reliability information available in *Dyslexia Differential Diagnosis*; 3DM, Blomert and Vaessen, 2009) was individually administered. The scores of the following 3DM subtests were used. *Word Reading task*: contains visually presented high-frequency words, low-frequency words and pseudowords. Accuracy (% correct) and fluency (correct words in 1 min) were measured. *Rapid automatized naming (RAN):* blocks of letters or numbers are presented and items have to be read as fast and accurate as possible. Fluency is the time in seconds needed to name a screen of 15 items. *LSS association tasks*: consist of identification and discrimination tasks. In the identification task an aurally presented speech sound has to be matched to one out of four visually presented letters. In the discrimination task the child has to judge whether the speech sound and letter on the screen are congruent or incongruent. *Computerized Spelling:* words are aurally presented and visually displayed on screen with missing letters. The participants have to select the missing letter out of four alternatives. For the last two subtests, accuracy (% correct) as well as response time (sec/item) is measured.

Finally, the *RAVEN Colored Progressive Matrices* was used to obtain an estimate of fluid IQ (RAVEN CPM; Raven et al., 1998) and the Child Behavior Checklist (CBCL) was completed by the parents to exclude any additional behavioral problems (Achenbach et al., 2003).

The group differences in reading accuracy and speed measures are displayed in **Table 1**. The table shows a deficit in dyslexics that is mainly manifested by large differences in the reading fluency measures, while both groups attained reasonably high levels of accuracy.

### ERP MEASUREMENT

#### Procedure and equipment

The ERP measurements were taken within a period of around 2 months. The EEG recording took place in a video-controlled and soundproof room with temperature regulated by an air-conditioning system. There was no exposure to sunlight and the lightning of the room allowed a uniform and glare-free illumination. Participants and lab assistants were together at all times in the room while the experimenter controlling the recording, subject performance and stimuli presentation was in an adjacent room. Participants were seated at approximately 80 cm distance from the computer screen and the lab assistant sat behind at a distance that safely avoided any possible distraction or interference on the visual field of the participant. At both arms of the participant’s chair response buttons were placed. The experiment lasted around 16 min including pauses, and it was part of a longer experimental session (around 2 h long). There were short pauses between blocks and longer breaks (around 5 min long) between experiments. The length of these pauses and breaks varied according to the needs of the participants and all of them received a present at the end of the experimental session. The stimuli were presented using an ASUS VW22U (resolution 1680 × 1050) monitor with a Dell Optiplex 760 dual-core 3.0 GHz computer and an ATI HD 6570, 2 Gb graphic card. The software used to present the stimuli was Presentation (Version 14.4, www.neurobs.com).

**FIGURE 1:**
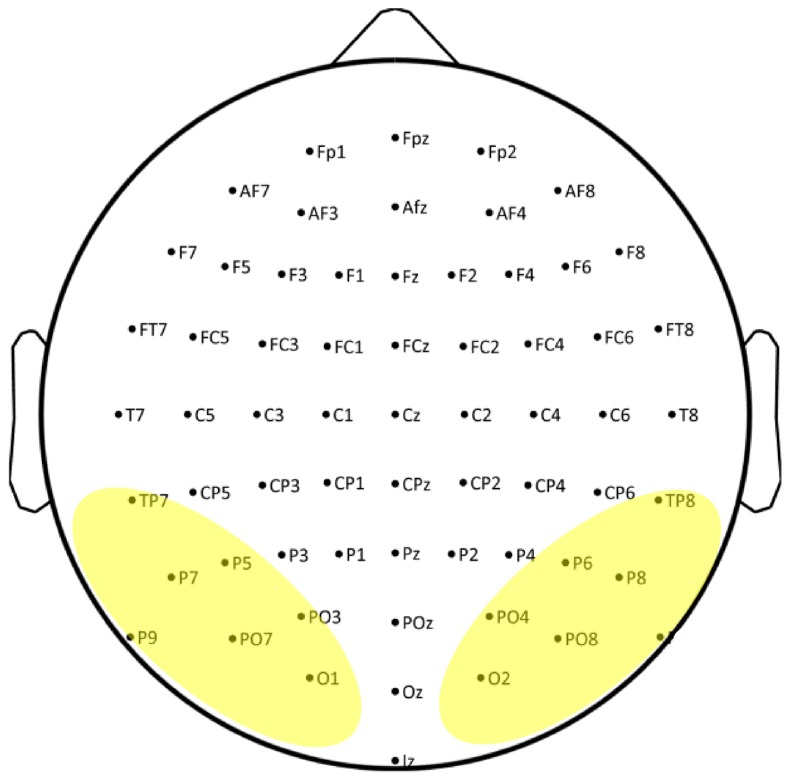
**Electrode sites across the scalp used in the current study.** Electrodes used in the analyses are indicated by highlighting.

The ERP data were collected using a 64 channels Biosemi ActiveTwo system (Biosemi, Amsterdam, Netherlands). EEG was recorded DC (low-pass: 5th order sync digital filter) with a 1024 Hz sample rate. The Biosemi system uses two additional electrodes (Common Mode Sense [CMS] and Driven Right Leg [DRL]) as recording reference and ground (see www.biosemi.com/faq/cms&drl.htm for details). The 64 electrodes were distributed across the scalp according to the 10–20 International system and applied using an elastic electrode cap (Electro-cap International Inc.). Electrode sites across the scalp are presented in **Figure 1** and the electrodes used in the analyses are indicated by highlighting. In addition, six external Flat-Type Active electrodes were used, four of which recorded vertical and horizontal electro-oculogram (EOG) and two were placed at mastoids for off-line reference.

#### Stimuli

Strings of words and symbols were used in the experiment. 80 bi-syllabic Dutch words were selected using estimates of age of acquisition (AOA). Our AOA criterion was 6 years or earlier. Estimates of AOA were based on two published ratings (1) vocabulary estimates of 6-year-olds (Schaerlakens et al., 1999), (2) AOA of Dutch words (Ghyselinck et al., 2000), and a subsequent student/parent familiarity rating of the selected words. The current selection criterion was motivated by a study indicating that AOA is a more sensitive index of lexical familiarity than either word frequency or neighborhood density when examining developmental change in visual word recognition (Garlock et al., 2001). Short vs. long strings contained four or five letters and long strings contained six and seven letters. 80 symbol strings were created by converting the previous words into a special font: “3elementSymbols-1600” (P.L. Cornelissen, personal communication October 2011) with similar number of line elements and comparable spatial frequency and contrast characteristics to actual letters (Pammer et al., 2004). To avoid symbols resembling the fixation cross, the letters “z” and “y” were replaced by “s” and “u” in the symbol strings. Short vs. long strings contained four or five characters and long strings contained six and seven characters.

#### Experimental design and task

**FIGURE 2:**
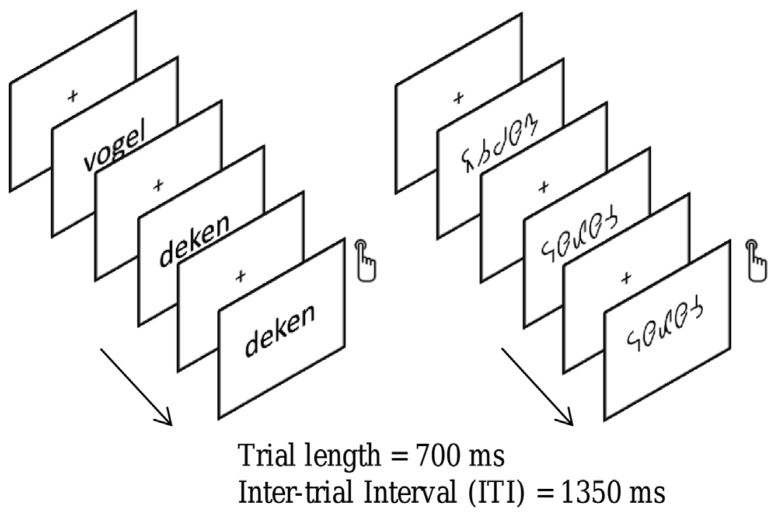
**An illustration of the word and symbol strings used in the present study.** Children were required to attend to the strings and to depress a button whenever a string was identical to its immediate predecessor. Strings of words and letter-like symbols were presented in a blocked design. A fixation cross was presented in between strings.

All stimuli were presented at the center of the screen with a visual angle subtending on average 1.5° × 6.4° (height × width), using the lower case font “Arial” in white on a black background, at a font size of 40 and bold. They were presented during 700 ms followed by a 1350 ms inter-stimulus interval (ISI) during which a white centered fixation cross was displayed. Blocks comprised 44 trials, four of which were target trials (i.e., immediate repetitions). The experiment had a 2 × 2 design with the experimental conditions String Length (short vs. long) and String type (word vs. symbol) evenly distributed in eight trial blocks. Four word and four symbol blocks alternated pseudo-randomly across participants. The presentation of the targets was pseudo-randomized to avoid consecutive presentations of targets. The participants were instructed to press a button when they detected a target (i.e., when a stimulus was immediately followed by itself). An example of the stimuli used and a schematic of the design are shown in **Figure 2**.

#### ERP preprocessing

All EEG data were preprocessed and analyzed with EEGLAB v.11.0.0.0b (Delorme and Makeig, 2004), an open source toolbox for Matlab (Mathworks, Inc.). When imported to EEGlab, the data were referenced to average mastoids, digitally filtered using a basic FIR filter (high pass 1 Hz and low pass 70 Hz), resampled to 256 Hz and epoched (from –500 to 1550 ms after stimulus onset). The baseline of each epoch was then corrected to remove residual activity differences prior to stimuli (this is done by subtracting the mean prestimulus activity from the waveform for each channel and epoch). Artifact removal was done in two steps. The first step consisted of visual inspection of the epochs to remove those epochs containing non-stereotyped artifacts such us head or muscle movements. Secondly, an independent component analysis (ICA) was run using the “runica” algorithm available in EEGlab (Makeig et al., 1997). The extended option was used to perform a version of the infomax ICA algorithm (Lee et al., 1999) that results in a better detection of sources with sub-Gaussian distribution, such as line current artifacts and slow activity. The resulting 64 ICA components were pruned by visual inspection of their scalp map, time course and mean activity, in order to remove components related to artifacts like line noise, eye blinks and ocular movements. The data was then reconstructed on an average (SD) of 34.75 (4.73) ICA components in the control group, and 32.32 (5.60) components in the dyslexic group. Spline interpolation was applied to channels with excessive artifacts. P9 and P10 were interpolated for only one participant. After artifact removal by ICA a new baseline correction was done. Afterwards, data were low pass filtered to 30 Hz (48 dB/octave) and re-referenced to the average of the 64 scalp electrodes. Trials with responses (i.e., target trials and false alarms) were not included in the statistical analysis. The mean (SD) number of trials included in the analysis (after removal of artifacts and response epochs) in the typical readers group, for short words, long words, short symbols and long symbols were 78.95 (1.79), 78.95 (1.27), 73.90 (3.40), and 73.2 (4.11), respectively. The mean (SD) number of trials included in the analysis in the dyslexic group for short words, long words, short symbols and long symbols were 78.63 (1.12), 77.79 (2.22), 75.74 (2.76), and 72.00 (6.09), respectively. Finally, individual subject averages were calculated for each experimental condition.

### STATISTICAL ANALYSIS

A repeated measures mixed-model ANOVA statistical analysis was performed comparing typical vs. dyslexic readers (between subjects factor *Dyslexia*). The within subjects factors defined in the analysis were the following. *String Type* (2 levels: words or strings of letter-like symbols); *String Length* (2 levels: short or long strings). *Hemisphere* (2 levels: right and left hemisphere); *Electrode* (7 levels. Electrodes pairs at occipital, occipito-temporal and parietal locations were included; O1–O2, PO7–PO8, PO3–PO4, TP7–TP8, P9–P10, P7–P8, P5–P6). Peaks were detected by searching for the maximum amplitude value within the time ranges of 50–180 ms for P1, 175–300 ms for N1, and 250–400 ms for P2. The peak values of amplitudes (μV) and latencies (ms) were used in analysis. Greenhouse–Geisser correction of degrees of freedom was used to calculate *p* values when the assumption of sphericity was violated.

In order to assess the relation between the N1 effect and reading fluency the left hemisphere sites (TP7, P9, P7, P5, PO7, PO3, and O1) were selected and averaged based on their proximity to the VWFA location. A composite score of word reading fluency was computed by averaging the One-Minute Test score and the 3DM word reading scores for high frequency and low frequency words. These three scores were combined because they are all based on single-word reading within one minute time and are, arguably, related to visual word recognition processes. Other fluency measures used in the behavioral tests were not included in the composite score since they may be sensitive to different processes (i.e., grapheme-phoneme conversion) and some use different stimuli (i.e., single letters or pseudowords). A linear regression analysis was then performed, for both groups, separately, between the N1 word-symbol difference in amplitude and the composite word fluency score.

## RESULTS

### EXPERIMENTAL TASK PERFORMANCE

#### Accuracy

The performance accuracy data were not normally distributed. Thus, Wilcoxon Signed-ranks test was performed to examine the differences between string type and string length, and independent samples Mann–Whitney-U test was performed to examine differences between groups.

The percentage of correct responses (button presses to targets) was significantly larger for words relative to symbol strings, *Z* = 5.02, *p* < 0.001. The mean (SD) percentages of correct answers to words and symbol strings were 83.65 (15.35) and 58.33 (18.99), respectively. The mean rank in favor of words was 19.53 while the mean rank in favor of symbol strings was 9.75. The percentages did not significantly differ between short and long strings, *p* = 0.632. With regard to the group differences, the percentage of correct responses was significantly larger in typical readers relative to dyslexics, for short words (*U* = 114, *p* = 0.033), and for long words (*U* = 116, *p* = 0.038). The percentages for short and long words were 89.37 (17.33) and 90 (9.60), for typical readers; and for dyslexics they were 78.29 (20.77) and 76.31 (21.20), respectively. Finally, the percentage of correct responses was larger in typical readers relative to dyslexics for long symbol strings, *U* = 110, *p* = 0.024, but not for short symbol strings, *p* = 0.095. The percentages for long symbol strings in typical readers and dyslexics were 66.87 (15.85) and 53.95 (17.70), respectively.

The percentage of false alarms (button presses to non-target stimuli) was larger for symbol strings than for words, *Z* = 5.32, *p* < 0.001. The mean (SD) percentage of false alarms to words was 1.10 (1.56) and to symbol strings 9.07 (4.74). The mean rank in favor of symbols was 19.92 while the mean rank in favor of words was 4.00. The percentage did not differ between short and long words, *p* = 0.418, but it was larger for long relative to short symbol strings, *Z* = 2.71, *p* = 0.007. The mean (SD) percentages for short and long symbol strings were 7.37 (4.69) and 10.77 (7.35), respectively. The mean rank in favor of long symbol strings was 17.26 while the mean rank in favor of short symbols was 16.19. Dyslexics and typical readers did not significantly differ in the percentages of false alarms, *p*s > 0.095.

#### Reaction times

Reaction times (RTs) of correct responses to target stimuli were subjected to repeated measures ANOVA with the within-subject factors String Length and Sting Type, and the between-subject factor Dyslexia. The analysis yielded a significant main effect of String Type, *F*(1,37) = 33.93, *p* < 0.001, η^2^ = 0.48, indicating shorter RTs to symbol strings [553.49 (216.68) ms] than RTs to word targets [751.69 (192.32) ms]. Furthermore, there was a significant effect of Dyslexia, *F*(1,37) = 4.85, *p* < 0.034, η^2^ = 0.12, indicating faster RTs in dyslexics [611.30 (241.80) ms], relative to typical readers [691.83 (206.25) ms]. All other effects did not reach significance, *p*s > 0.163 The performance pattern suggests a group difference in speed-accuracy tradeoff; dyslectics responded faster than typical readers but made more errors.

### ERP RESULTS

#### P1 component

**FIGURE 3:**
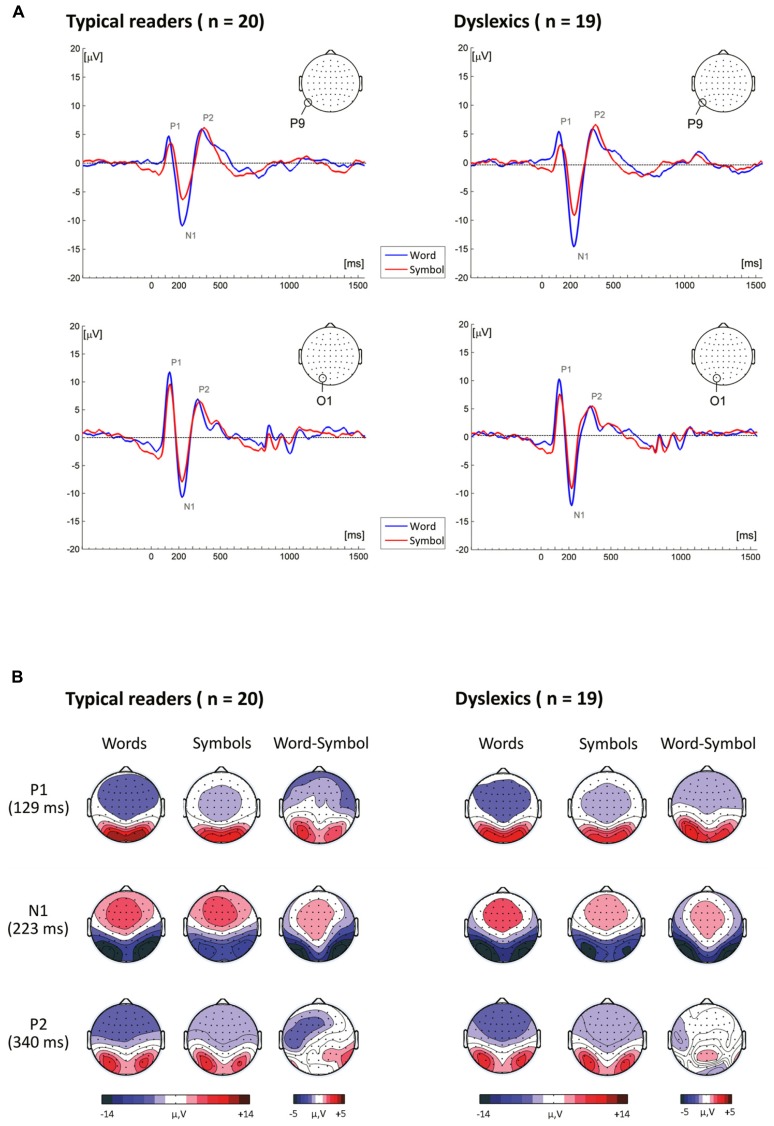
**(A)** Event-related brain potentials to word and symbol strings at P9 and O1. **(B)** Topographical maps showing the time course of neural activity following stimulus presentation.

***P1 amplitudes.*** The P1, peaking on average at around 127 ms, is presented in **Figure 3**. The ANOVA performed on P1 amplitudes included the within-subject factors String Type, String Length, Electrode and Hemisphere and the between-subject factor Dyslexia. The analysis yielded a significant main effect of String Type, *F*(1,37) = 46.27, *p* < 0.001, η^2^ = 0.56, indicating that P1 amplitudes were larger for words than for symbol strings. The mean (SD) amplitude for words was 6.89 (2.04) μV and for symbol strings 5.32 (1.92) μV. The main effect of String Type was qualified by an interaction with String Length *F*(1,37) = 4.32, *p* = 0.045, η^2^ = 0.10, indicating a larger Type effect for long relative to short strings. Moreover, String Type also interacted with Electrode, *F*(3,116) = 17.74, *p* < 0.001, η^2^ = 0.32, and Hemisphere, *F*(1,37) = 5.13, *p* = 0.030, η^2^ = 0.12. This interaction indicated that the String Type effect was more pronounced at the most posterior sites PO7–PO8, P7–P8 and O1–O2, and at the left relative to the right hemisphere sites. In addition, there was a significant interaction between String Length and Electrode, *F*(3,96) = 3.68, *p* = 0.019, η^2^ = 0.09, indicating that at PO3–PO4 and O1–O2 sites amplitudes were slightly larger for long relative to short strings.

**FIGURE 4:**
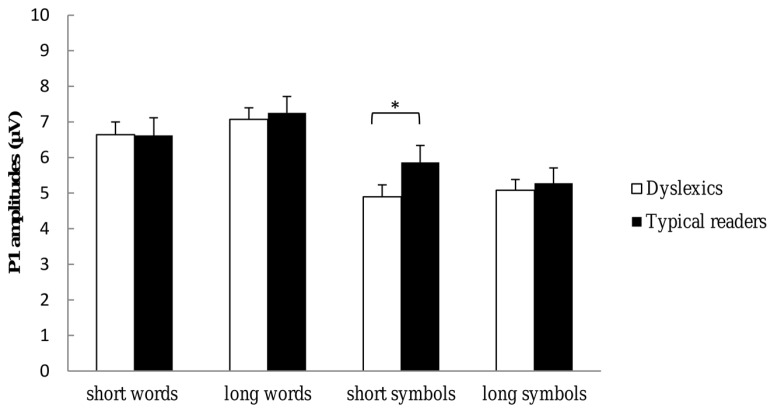
**P1 amplitudes to each of the string types and string lengths used in the present study.** Open bars refer to the P1 amplitudes of dyslexic readers and filled bars to the P1 amplitudes of typical readers. P1 amplitudes are averaged across TP7, TP8, P9, P10, P7, P8, P5, P6, PO7, PO8, PO3, PO4, O1, and O2 electrode sites. *p < 0.05.

Most importantly, there was a significant three-way interaction including Dyslexia, String Type and String Length, *F*(1,37) = 4.30, *p* = 0.045, η^2^ = 0.10, suggesting larger amplitudes for typical readers relative to dyslexics for short symbol strings but not for long symbol strings nor for words. This interaction is plotted in **Figure 4**. Finally, there was a higher-order interaction including Dyslexia, String Length, Hemisphere and Electrode, *F*(4,134) = 3.93, *p* = 0.006, η^2^ = 0.10. This interaction indicated that P1 amplitudes at the PO3–PO4 and O1–O2 sites were larger for typical readers relative to dyslexics, and this effect was more pronounced at left relative to right hemisphere sites and for short relative long strings (see follow-up analysis below). All other effects did not reach significance, *p*s > 0.159.

Follow-up analysis for symbol strings revealed a significant interaction between String Length and Dyslexia, *F*(1,37) = 4.58, *p* = 0.039, η^2^ = 0.11, showing larger amplitudes for typical readers relative to dyslexics to short symbol strings but not to long symbol strings (see **Figure 4**). This interaction was not significant for words, *p* = 0.481. Separate ANOVAs were performed for each hemisphere. The analysis for left hemisphere sites revealed a significant interaction between Dyslexia, String Length and Electrodes, *F*(3,123) = 2.67, *p* = 0.045, η^2^ = 0.07. This interaction indicated that amplitudes were larger at PO3 and O1 sites for typical readers relative to dyslexics, and this difference was more pronounced for short relative to long strings. The interaction was not significant for the right hemisphere sites, *p* = 0.515.

In short, the analysis of P1 amplitudes indicated that P1 amplitude is larger for words compared to symbol strings. This enhancement was more pronounced for long relative to short strings and at the most posterior sites over the left hemisphere. Finally, P1 amplitude was larger to short symbol strings in typical readers compared to dyslexics.

***P1 latencies.*** The ANOVA performed on P1 latencies yielded a significant main effect of String Type*, F*(1,37) = 16.63, *p* < 0.001, η^2^ = 0.31, indicating shorter latencies for word stimuli than for symbol strings. The mean (SD) latencies were 123.73 (9.00) ms, 130.19 (10.84) ms for words and symbol strings, respectively. The effect of String Type was qualified by an interaction with Electrode, *F*(4,142) = 3.66, *p* = 0.008, η^2^ = 0.09, indicating that the effect was primarily at the TP7–TP8 sites, and a higher-order interaction including Electrode, String Length and Hemisphere, *F*(3,112) = 4.67, *p* = 0.004, η^2^ = 0.11. The latter interaction showed that the effect of String Type at the right TP8 site was more pronounced for short relative to long strings, while at the left TP7 site the String Type effect was more pronounced for long relative to short strings. All other effects were not significant, *p*s > 0.178, although the main effect of Dyslexia approached significance, *F*(1,37) = 3.51, *p* = 0.069, η^2^ = 0.09, suggesting a trend for shorter latencies in dyslexics relative to typical readers. The mean (SD) latencies for typical readers and dyslexics were 129.42 (7.32) and 124.37 (9.43), respectively.

#### N1 component

***N1 amplitudes.*** A pronounced negativity, peaking at around 223 ms, is visible in the topographical maps presented in **Figure 3**. N1 amplitudes were submitted to ANOVA with the within-subjects factors String Type, String Length, Electrode and Hemisphere and the between-subjects factor Dyslexia. The analysis yielded a significant main effect of String Type, *F*(1,37) = 131.26, *p* < 0.001, η^2^ = 0.78, indicating larger amplitudes for words (12.15 (3.78) μV), relative to symbol strings (9.00 (3.48) μV). This effect was qualified by an interaction with Electrode, *F*(3,97) = 28.03, *p* < 0.001, η^2^ = 0.43, and a three-way interaction with String Length and Electrode, *F*(3,107) = 4.07, *p* = 0.010, η^2^ = 0.10, indicating that the String Type effect was more pronounced at P9–P10, P7–P8, PO7–PO8 and O1–O2 electrode sites, and larger for long strings.

The ANOVA also yielded a significant main effect of String Length *F*(1,37) = 4.55, *p* = 0.040, η^2^ = 0.11, indicating that N1 amplitudes were slightly larger for long relative to short strings, 10.75 (3.68) μV and 10.39 (3.46) μV, respectively. This effect was qualified by interactions with Electrode and Hemisphere, *F*(2,81) = 4.79, *p* = 0.009, η^2^ = 0.11, and *F*(1,37) = 13.86, *p* = 0.001, η^2^ = 0.27, respectively. These interactions indicated an effect of String Length that was more pronounced at the P7–P8, P5–P6, PO7–PO8 and PO3–PO4 electrode sites, and at the right relative to the left hemisphere sites.

**FIGURE 5:**
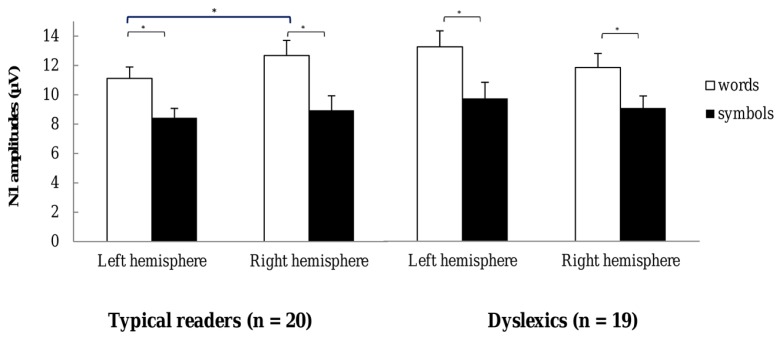
**N1 amplitudes for words and symbols recorded over the left and right hemisphere sites.** Open bars refer to N1 amplitudes for word strings and filled bars to the N1 amplitudes for symbol strings. The left hemisphere N1 amplitudes are averaged across the sites TP7, P9, P7, P5, PO7, PO3, and O1, and the right hemisphere amplitudes across homologue pairs. Plots represent N1 amplitudes in typical readers (left) and in dyslexics (right). *p < 0.05.

Most importantly, there was a significant three-way interaction including String Type, Hemisphere and Dyslexia, *F*(1,37) = 6.99, *p* = 0.012, η^2^ = 0.16. This interaction is plotted in **Figure 5**. The String Type effect was more pronounced in typical readers relative to dyslexics at the right hemisphere sites, while the String Type effect was less pronounced in typical readers relative to dyslexics at the left hemisphere sites (see follow-up ANOVAs below). Additionally, there was a three-way interaction between Dyslexia and Hemisphere that approached significance *F*(1,37) = 3.47, *p* = 0.070, η^2^ = 0.09, suggesting a trend for larger amplitudes in dyslexics relative to typical readers, at the left hemisphere sites but not at the right hemisphere sites. All other effects were not significant, *p*s > 0.094.

Follow-up ANOVAs were performed in both groups and string types, separately. The analysis for the typical readers data revealed that the String Type effect was reduced at the left hemisphere relative to the right hemisphere sites, *F*(1,19) = 5.54, *p* = 0.029, η^2^ = 0.23. The analysis for words revealed a main effect of Hemisphere, *F*(1,19) = 4.49, *p* = 0.048, η^2^ = 0.19, suggesting that the amplitude for words was reduced at the left hemisphere relative to the right hemisphere sites. The analysis for symbol strings did not show a significant main effect of Hemisphere, *p* = 0.512. In addition, the interaction including Hemisphere and String Length *F*(1,19) = 9.86, *p* = 0.005, η^2^ = 0.34, indicated that at the right hemisphere sites, long symbols elicited larger amplitudes relative to short symbols. In the dyslexics data none of the interactions with Hemisphere approached significance, *p*s > 0.158, indicating that neither symbol nor word N1 amplitudes differed across hemispheres.

To sum up, the analysis of N1 amplitudes revealed larger responses to words compared to symbol strings. This effect was more pronounced for long stimuli and at posterior sites. N1 amplitudes were also enhanced for long relative to short strings at the posterior right-hemisphere sites. Importantly, the analysis revealed that for typical readers, N1 word amplitudes were reduced at the left compared to the right hemisphere sites, but this effect was absent in dyslexics.

**FIGURE 6:**
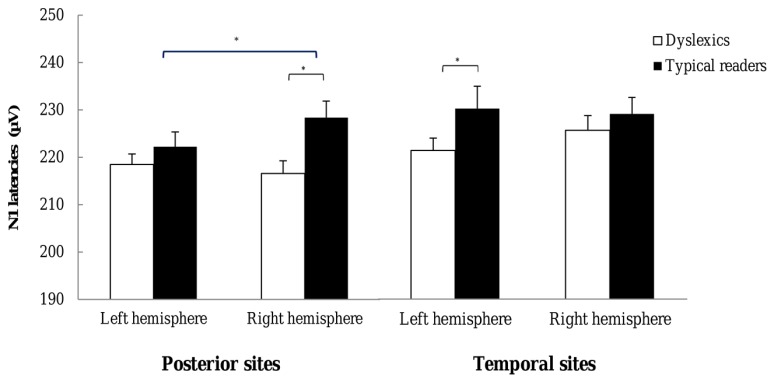
**N1 latencies observed for each hemisphere.** Open bars refer to the N1 latencies of dyslexic readers and filled bars to the N1 latencies of typical readers. For each plot latencies are averaged across the most posterior P7–P8, P5–P6, PO7–PO8, and O1–O2 pairs (left) and across the most temporal P9–P10 and TP7–TP8 electrode pairs (right). *p < 0.05.

***N1 latencies.*** The ANOVA on N1 latencies revealed an interaction between String Type and Hemisphere, *F*(1,37) = 5.47, *p* = 0.025, η^2^ = 0.13, indicating that latencies were longer for symbols than word strings but only at the right hemisphere electrode sites. N1 latencies at the right hemisphere for words and symbols were 222.81 (14.09) and 225.20 (14.28), respectively. The main effect of Dyslexia, just fell short of significance, *F*(1,37) = 4.06, *p* = 0.051, η^2^ = 0.09, but was included in a three-way interaction with Hemisphere and Electrode, *F*(3,128) = 3.02, *p* = 0.026, η^2^ = 0.07. This interaction indicated that the effect of Dyslexia was more pronounced at the right relative to the left hemisphere at the sites P7–P8, P5–P6, PO7–PO8, PO3–PO4, and O1–O2; while it was more pronounced at left relative to right hemisphere at sites TP7–TP8 and P9–10. The interaction is plotted in **Figure 6**. Finally, the interaction including String Type, Hemisphere and Electrode just fell short of significance, *F*(2,92) = 2.63, *p* = 0.065, η^2^ = 0.06. The String Type effect tended to be larger at the right TP8 and P10 electrodes, relative to their left hemisphere homologue pairs. All other effects were not significant, *p*s > 0.117.

#### P2 component

***P2 amplitudes.*** The P2 peaked at around 341 ms and is presented in **Figure 3**. The ANOVA performed on P2 amplitudes revealed a small but significant effect of String Type, *F*(1,37) = 4.95, *p* = 0.032, η^2^ = 0.12, indicating slightly larger positivities for words relative to symbol strings; 7.52 (2.73) μV and 7.03 (2.32) μV, respectively. This effect was qualified by interactions with Electrode and Hemisphere, *F*(3,114) = 5.61, *p =* 0.001, η^2^ = 0.13, and *F*(1,37) = 5.04, *p* = 0.031, η^2^ = 0.12, respectively. In addition, there was a three-way interaction of String Type, Electrode and Hemisphere, *F*(4,146) = 3.97, *p* = 0.005, η^2^ = 0.10, showing that the word-related enhancement was more pronounced at P9–P10, P7–P8, P5–P6 and PO3–PO4 sites, and larger at the right relative to the left hemisphere sites (see additional ANOVAs below). The main effect of Hemisphere was also significant, *F*(1,37) = 5.34, *p* = 0.026, η^2^ = 0.13, indicating that amplitudes were larger at the left relative to the right hemisphere sites. P2 amplitudes for left and right hemisphere sites were 7.73 (2.89) and 6.82 (2.54), respectively. Moreover, there was a significant effect of String Length in interaction with Electrode, *F*(1,90) = 6.40, *p* = 0.001, η^2^ = 0.15, indicating larger amplitudes for long relative to short strings at PO3–PO4 and O1–O2 sites.

**FIGURE 7:**
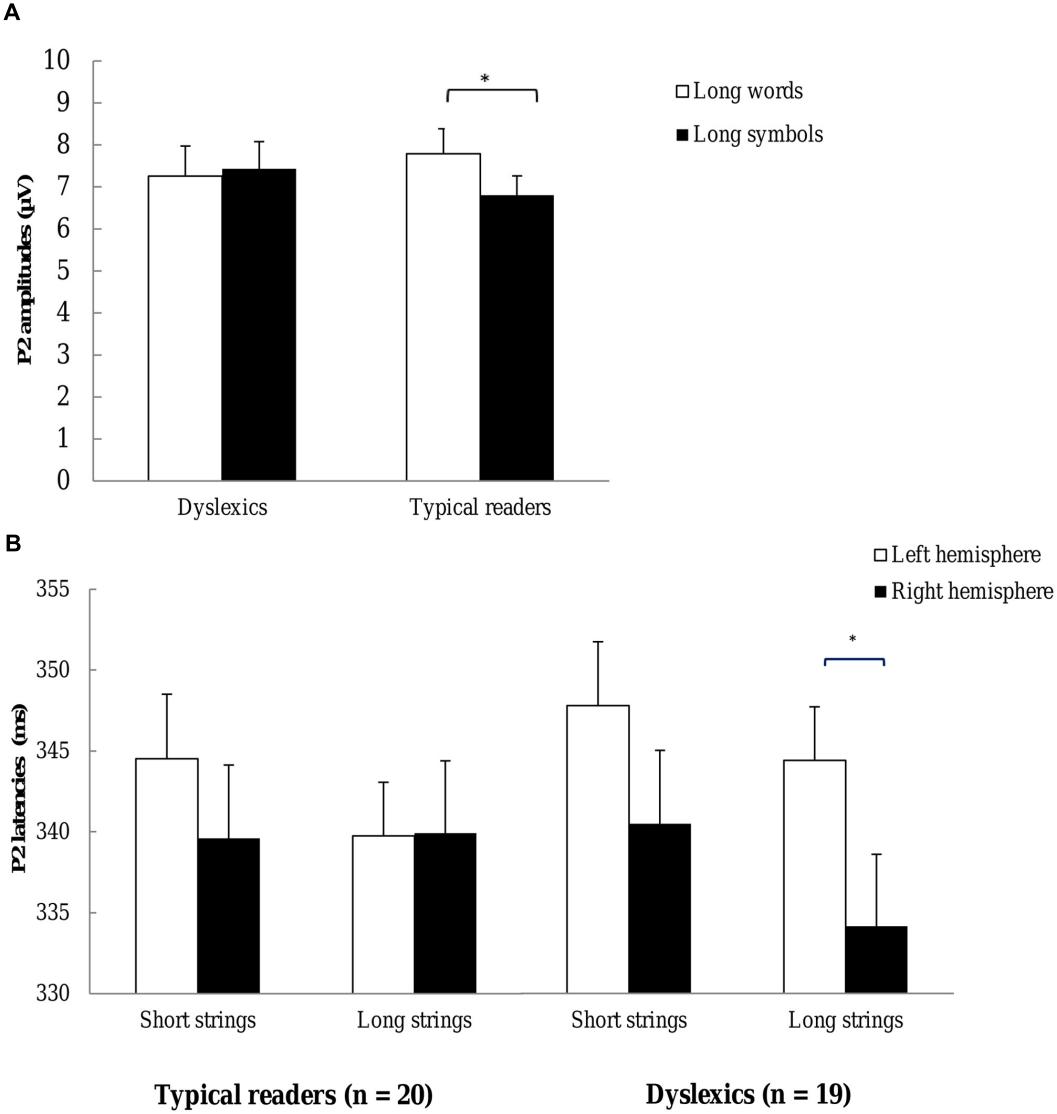
**P2 (A) P2 amplitudes for each group.** Open bars refer to the P2 amplitudes to words and filled bars to the P2 amplitudes to symbol strings. Plots represent P2 amplitudes to long strings. P2 amplitudes are averaged across TP7, TP8, P9, P10, P7, P8, P5, P6, PO7, PO8, PO3, PO4, O1, and O2 electrode sites. **(B)** P2 latencies to each string length. Open bars refer to the P2 latencies at left hemisphere sites and filled bars to P2 latencies at right hemisphere sites. Plots represent P2 latencies of typical readers (left) and dyslexics (right). *p < 0.05.

Most importantly, there was a three-way interaction including Dyslexia, String Type and String Length, *F*(1,37) = 6.48, *p* = 0.015, η^2^ = 0.15. This interaction showed that the String Type effect was larger in typical readers relative to dyslexics for long strings but the groups did not differ in the String Type effect for short strings. This interaction is plotted in **Figure 7A**. Finally, a three-way interaction between String Type, String Length and Electrode approached significance *F*(3,100) = 2.47, *p* = 0.072, η^2^ = 0.06, indicating a trend for a larger String Type effect associated with short relative to long strings. All other effects were not significant, *p*s > 0.133.

Additional ANOVAs were performed for each String Length and String Type. The analysis for long strings revealed a significant interaction between String Type and Dyslexia, *F*(1,37) = 5.87, *p* = 0.020, η^2^ = 0.14, suggesting that the String Type effect was larger in typical readers relative to dyslexics. The analysis for short strings did not show significant interactions including String Type and Dyslexia, *p* = 0.608. Finally, the interaction between String Length and Dyslexia just fell short of significance for symbol strings, *F*(1,37) = 3.97, *p* = 0.054, η^2^ = 0.10, but did not approach significance for words, *p* = 0.173. P2 amplitudes for long symbol strings tended to be smaller for typical readers relative to dyslexics (see **Figure 7A**).

The current analysis of P2 amplitudes revealed a small but significant enhancement to words relative to symbol strings. This effect appeared to be larger at right posterior sites compared to left sites. Moreover larger amplitudes for long relative to short strings were observed at occipital sites. Importantly, typical readers showed larger P2 responses for long words relative to long symbols relative to dyslectics but this effect was absent for short strings.

***P2 latencies.*** For P2 latencies, there was a significant effect of String Type, *F*(1,37) = 25.48, *p* < 0.001, η^2^ = 0.41, indicating that words elicited faster P2 responses than symbol strings, 337.28 (16.51) ms and 345.40 (16.11) ms, respectively. This effect was qualified by an interaction with Electrode, *F*(4,151) = 4.03, *p* = 0.004, η^2^ = 0.10, suggesting that the effect was less pronounced at PO3–PO4 and O1–O2 sites. In addition, there was a main effect of Hemisphere, *F*(1,37) = 5.78, *p* = 0.021, η^2^ = 0.13, indicating that latencies were longer at the left relative to the right hemisphere sites. Latencies for left and right hemisphere sites were 344.09 (17.96) ms and 338.58 (16.37) ms, respectively. Furthermore, there was a main effect of String Length, *F*(1,37) = 6.58, *p* = 0.014, η^2^ = 0.15, suggesting that P2 peaked later for short compared to long strings; latencies were 343.09 (15.54) ms and 339.59 (16.69) ms, respectively.

Importantly, the ANOVA yielded a significant three-way interaction between Dyslexia, String Length and Hemisphere, *F*(1,37) = 4.97, *p* = 0.032, η^2^ = 0.12. This interaction is plotted in **Figure 7B**. This interaction indicated shorter latencies at the right relative to the left hemisphere for long strings in dyslexics, but not for typical readers. Finally, the interaction between String Type, Electrode and Dyslexia approached significance, *F*(4,151) = 2.24, *p = .*066, η^2^ = 0.06, showing a trend for a more pronounced String Type effect in typical readers relative to dyslexics, at PO3–PO4 and O1–O2 sites. All other effects were not significant, *p*s > 0.163.

Additional ANOVAs were performed for each String Length. The analysis for long strings showed a significant interaction between Dyslexia and Hemisphere, *F*(1,37) = 4.70, *p =* 0.035, η^2^ = 0.11. The effect of Hemisphere was significant in dyslexics, *F*(1,18) = 9.34, *p =* 0.007, η^2^ = 0.34, but not in typical readers, *p* = 0.961. More specifically, latencies were shorter at the right relative to the left hemisphere sites for dyslexics but not for typical readers. The analysis for short strings did not reveal significant interactions with Dyslexia and Hemisphere, *p*s > 0.640.

### RELATION TO READING FLUENCY

**FIGURE 8:**
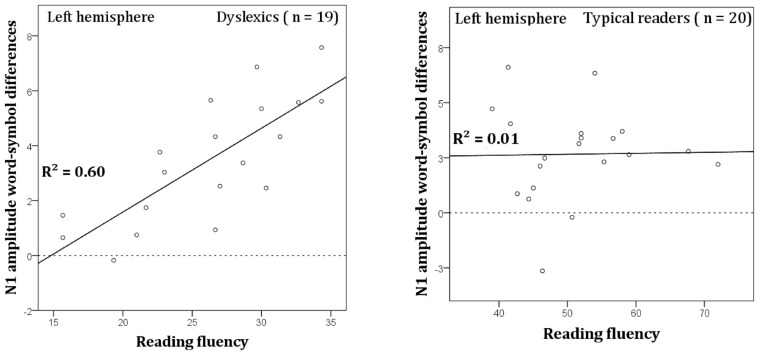
**Correlation between N1 word-symbol amplitudes averaged across the left hemisphere sites TP7, P9, P7, P5, PO7, PO3, and O1, and the composite reading fluency scores for dyslexic (left) and typical readers (right)**.

The word vs. symbol difference in N1 amplitude was computed and averaged across the left hemisphere electrode sites (TP7, P9, P7, P5, PO7, PO3, and O1). These difference scores were then submitted to regression analysis to assess the relation with reading fluency in dyslexic readers. An estimate of reading fluency was obtained by using the number of correctly read words per minute (composite score of three word reading tasks, see Statistical Analysis). The relation between N1 amplitude and reading fluency was significant, *R* = 0.78, *R*^2^ = 0.60, *β* = 1.97, *t* = 5.07, *p* < 0.001, and is plotted in **Figure 8**. It can be seen that faster dyslexic readers showed a more pronounced difference in N1 amplitude between words vs. symbols. A similar analysis was performed on the data obtained from the normal readers but this analysis did not show a significant relation between the N1 amplitude difference and reading fluency (see **Figure 8**).

## DISCUSSION

The present study obtained significant evidence for word specialization in early ERP components, as revealed by P1 and N1 amplitudes, at posterior temporo-occipital and parietal sites. N1 amplitudes were larger for words relative to symbol strings in both groups. Most interestingly, N1 amplitudes to words were smaller at the left compared to the right hemisphere sites in typical readers but not in dyslexics. Furthermore, the difference in N1 amplitude between words and symbols observed over the left hemisphere was related to reading fluency in the dyslexic group. Collectively, this pattern of results supports the notion that N1 amplitude is sensitive to visual word specialization (Bentin et al., 1999; Maurer et al., 2005) and discriminates between typical readers and dyslexics (Dujardin et al., 2011; Maurer et al., 2011).

The major focus of the current study was the sensitivity of the N1 component to word reading in normal and dyslexic readers. The results showed that N1 amplitude was sensitive to string length; i.e., longer strings elicited larger N1 amplitudes irrespective of the type of string. This may suggest that long strings imposed greater processing demands than short strings. As anticipated, there was also a pronounced effect of string type on N1 amplitude. That is, N1 amplitude was larger for words compared to symbol strings. This effect was found in both typical and dyslexic readers and can be interpreted in terms of N1 sensitivity to visual expertise and familiarity. The N1 enhancement for words vs. false fonts in children with dyslexia at the beginning of grade three was also reported in a previous study (Hasko et al., 2013) suggesting that there is some degree of print sensitivity at this level of reading experience also in dyslexic readers. In this study, both dyslexic and typical readers, already have an advanced level of letter knowledge (3rd grade). Indeed, the current behavioral results indicate that accuracy of the dyslexic children is reasonably high on most of the reading tasks. Their deficit is manifested primarily in reading fluency. A fluency deficit seems to be a common finding in dyslexia studies involving languages with relatively shallow orthographies such as German or Dutch (Landerl et al., 1997; Frith et al., 1998; Paulesu et al., 2001). Thus, in view of the level of expertise, some degree of low-level visual specialization for print in both groups can be expected. In typical readers, this is further supported by longitudinal data indicating that the majority of children show a distinct N1 for words relative to symbols already in 2nd grade (Maurer et al., 2006).

Importantly, in typical readers N1 amplitude for words was reduced at the left hemisphere compared to the right hemisphere. This hemispheric difference was not present in the dyslexic group. In this regard, there is an apparent discrepancy between the current results and previous findings showing a reduced difference between the N1 to words vs. symbols for reading impaired relative to normal readers (Helenius et al., 1999; Maurer et al., 2007; Kronschnabel et al., 2013). This discrepancy can be interpreted in various ways.

One interpretation refers to VWFA specialization. The lower N1 word amplitudes at the left hemisphere sites in typical readers relative to dyslexics might reflect facilitated lexical access. Lower N1 amplitudes in relation to higher frequency words that are easier to retrieve have been reported previously (Assadollahi and Pulvermüller, 2003; Hauk and Pulvermüller, 2004; Kronbichler et al., 2007). Thus, typical readers might have benefited from a whole-word level of specialization for the current word strings (all familiar words). This beneficial effect might be less pronounced in dyslexics, as suggested by the behavioral word reading scores showing that dyslexics underperformed on the word reading tests. On the experimental task, dyslexics also were less accurate than typical readers when words were presented, suggesting a deficit in whole-word level specialization. The interpretation based on the VWFA word-level specialization is supported by fMRI studies reporting a lack of word familiarity effect in VWFA activation in dyslexia (van der Mark et al., 2009), and an increased engagement of visual occipital areas relative to non-impaired readers (Wimmer et al., 2010). A reduced left-lateralized activation of occipito-temporal areas, as current results suggest in typical readers, might correspond to more automatized reading (Maurer et al., 2006) or to a level of higher expertise at which the facilitation from phonological and semantic areas may become more efficient (Price and Devlin, 2011). Furthermore, longitudinal studies in typical readers suggest that N1 amplitudes to print-specific stimuli are larger and more bilateral in 2nd grade compared to adults (Maurer et al., 2006). A similar decrease in N1 amplitude has been reported from 2nd to 5th grade in typical readers while an opposite trend was observed in dyslexics (Maurer et al., 2011).

An alternative interpretation assumes that attentional strategies might have contributed to the group differences in N1 amplitudes. Deficits in visual-spatial attention processing in dyslexia have been reported in previous behavioral studies (Facoetti et al., 2000; Valdois et al., 2004). Accordingly, reduced sensitivity has been shown in dyslexic children required to detect small changes in false-font symbol strings (Pammer et al., 2004). The lower rate of correct responses to symbol strings in dyslexics observed in the current experiment might then be a manifestation of deficient or deviant allocation of visual attention resources to the strings presented during the task. Furthermore, the higher percentage of false alarms and lower rate of correct responses to symbol strings compared to words suggest increased task demands associated with symbol strings. Attention modulation of early ERPs has been previously reported in the literature (see review in Luck et al., 2000). Stronger N1 responses have been observed in relation to stimuli presented at attended relative to unattended locations (see reviews in Luck et al., 2000; Vogel and Luck, 2000), and the interaction of attentional systems and the VWFA have been previously reported (Vogel et al., 2012). In the current study, relative to normal readers, dyslexics might have allocated more attention to word strings. This interpretation is consistent with the deficits manifested by the performance on word reading tests (see **Table 1**) and with the performance on the experimental task showing a lower rate of correct responses for words and shorter RTs in dyslexic compared to typical readers. The allocation of more attention to word strings is likely to result in a more pronounced activation of VWFA, thus enhancing N1 activation for words in dyslexics relative to typical readers. In this context, dyslexic children might have relied more strongly than typical readers on orthographic rather than phonological or semantic information. This could contribute to enhanced N1 amplitudes, as it has been previously reported that attention allocation to orthography evoked larger negativity compared to a semantic or phonological focus of attention (Ruz and Nobre, 2008). This interpretation is supported by a study reporting left fusiform activation that is inversely related to word-likeness of visually presented stimuli in a one-back task (Wang et al., 2011). These findings have been interpreted to suggest increased pressure on the visual system relating to higher short-memory demands imposed by stimuli lacking semantic or phonological information.

In the current study, we obtained a relation between word reading fluency scores (number of correct words read in a minute) and N1 amplitude enhancement for words at left hemisphere sites in dyslexic readers. While other studies collapsed groups of typical and dyslexic readers (Maurer et al., 2006, 2007), the current study showed this relation for dyslexic children but not for typical readers. N1 word amplitudes have been previously related to faster reading in unimpaired readers (Korinth et al., 2012). Collectively, the current findings suggest a stronger reliance on visual processing in dyslexics, which might be comparable to typical readers during earlier stages of reading acquisition. This is in accordance to longitudinal studies suggesting that N1 word-specific responses progressively decline after the first years of reading acquisition (Maurer et al., 2006). In this regard, the faster dyslexic readers might have benefitted from a stronger allocation of attentional resources to visual orthographic cues, which would also be consistent with attentional modulation of N1 amplitude, as discussed previously.

Finally, although not the target components of the present study, the P1 and P2 appeared to discriminate between groups. The P1 amplitudes to short strings at left occipital sites, and to short symbol strings across all sites, were larger for typical readers than for dyslexics. This pattern of findings might suggest that proficient readers co-activated letter representations to detect repetitions in short symbol strings, resulting in larger P1 amplitudes. P2 amplitudes for long strings did not discriminate between words and symbols in dyslexics but they did so in typical readers. Moreover, P2 latencies for long strings were shorter over the right relative to the left hemisphere in dyslexics but did not differ across hemisphere in typical readers. This difference might suggest facilitated access and, possibly, a more efficient allocation of attentional resources between typical and dyslexic readers. This interpretation is supported by studies showing that ERP positivities, peaking around 300 ms, are associated with improved performance on tasks using visual stimuli (Wickens et al., 1983; Nenert and Chaix, 2010).

## CONCLUSION

The present results provide evidence for differences in N1 word specialization between dyslexic and typical readers. Both groups showed N1 enhancement for words vs. symbol strings, but in typical readers the N1 amplitude for words was reduced over the left relative to the right hemisphere sites. This effect was absent in dyslexic readers. The current study differed from previous research with regard to the symbol strings used to assess the efficiency of word processing. The pattern of results suggests that the symbol strings used in this study might provide a sensitive tool for assessing N1 word specialization in dyslexic readers. The relation observed between the N1 word-specific amplitudes and reading speed measurements in the dyslexic children provides further support for this sensitivity. The current findings, suggesting a deficit at the level of visual word specialization in dyslexics, should be followed up by a longitudinal analysis to assess whether the apparent deficit in visual word specialization in dyslexic children decreases when they attain higher levels of reading fluency (e.g., following a remediation program).

## Conflict of Interest Statement

The authors declare that the research was conducted in the absence of any commercial or financial relationships that could be construed as a potential conflict of interest.
